# Supplementary material for: Rainfall and sea level drove the expansion of seasonally flooded habitats and associated bird populations across Amazonia
Source: Nat Commun. 2022 Aug 23;13:4945. doi: 10.1038/s41467-022-32561-0 (PMC9399099; doi:10.1038/s41467-022-32561-0)
Supplement: Supplementary file 1 — Supplementary Information [file 41467_2022_32561_MOESM1_ESM.pdf]

Rainfall and sea level drove the expansion of seasonally flooded habitats and associated bird populations across Amazonia

Supplementary Figures

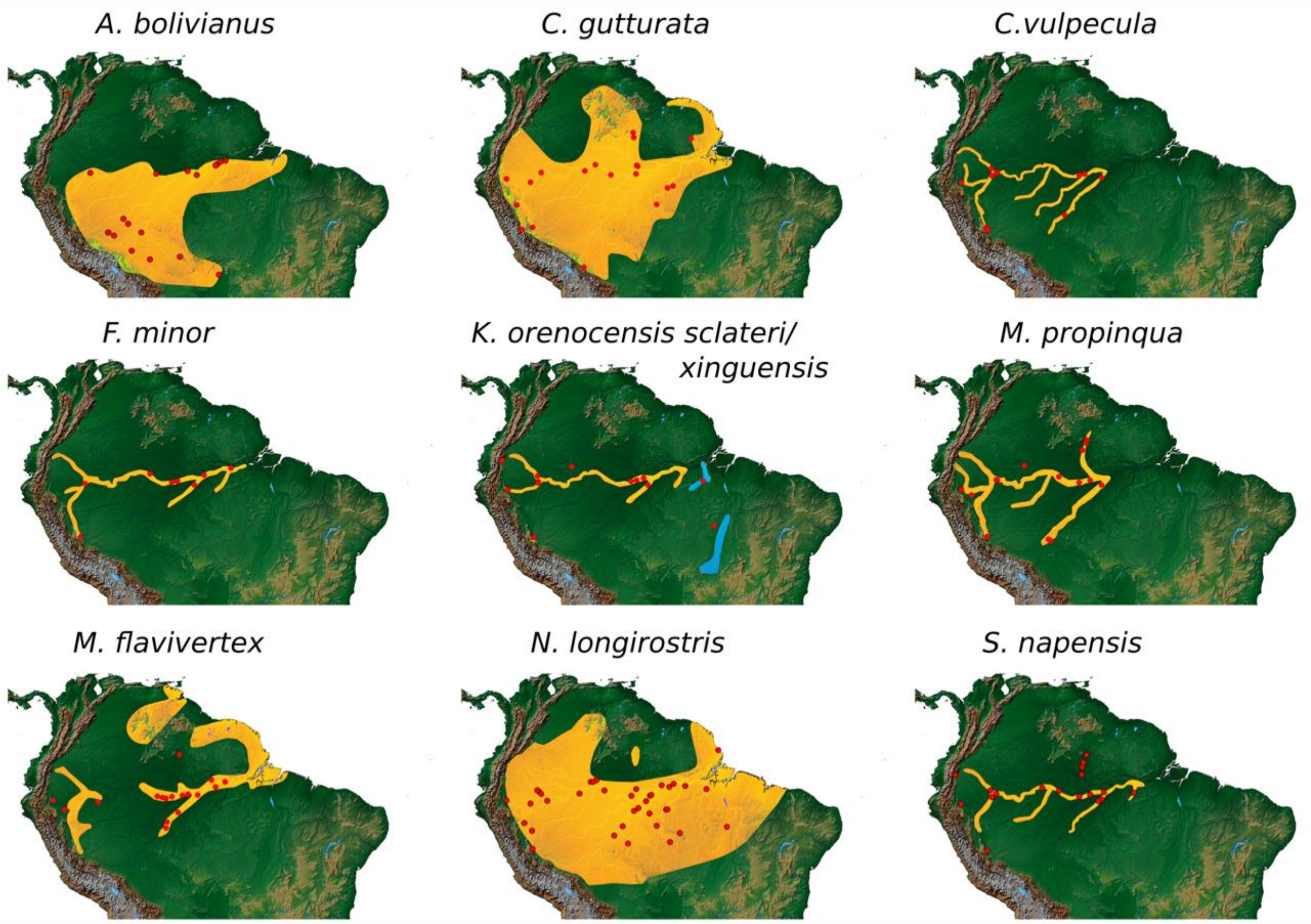

Supplementary Figure 1. Geographical distribution of the studied bird species used to reconstruct population demography. In each map, red dots depict the localities of the samples used in this study and yellow shades represent the described distribution of the species according to the International Union for Conservation of Nature (IUCN, iucnredlist.org/). For *K. orenocensis*, yellow and blue shades depict the described distribution of subspecies *K. o. sclateri* and *K. o. xinguensis*, respectively.

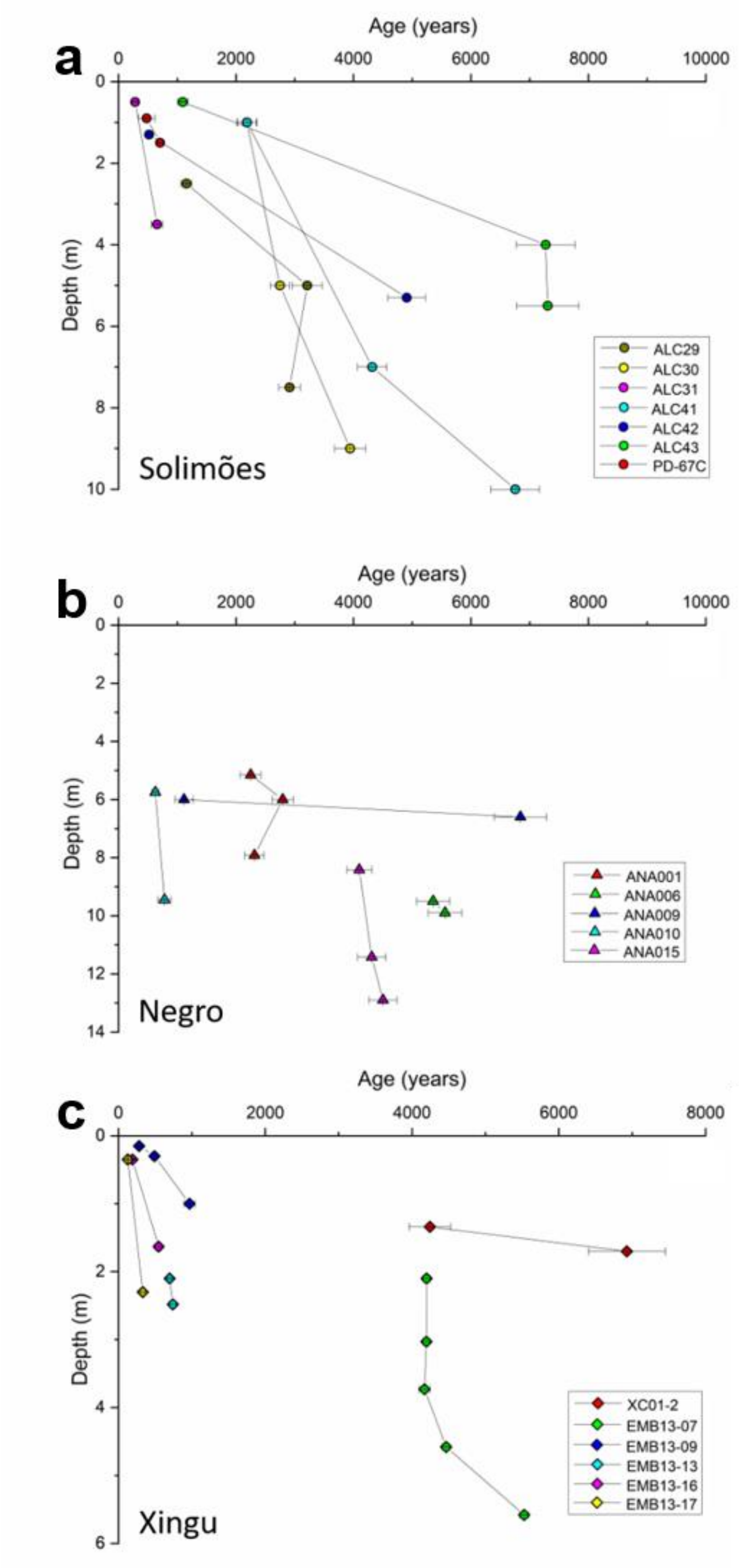

Supplementary Figure 2. Age-depth profiles used for calculation of sedimentation rates of seasonally flooded substrates of the Solimões (a), Negro (b) and Xingu (c) rivers. Age and depth data are presented in Supplementary Data 3.

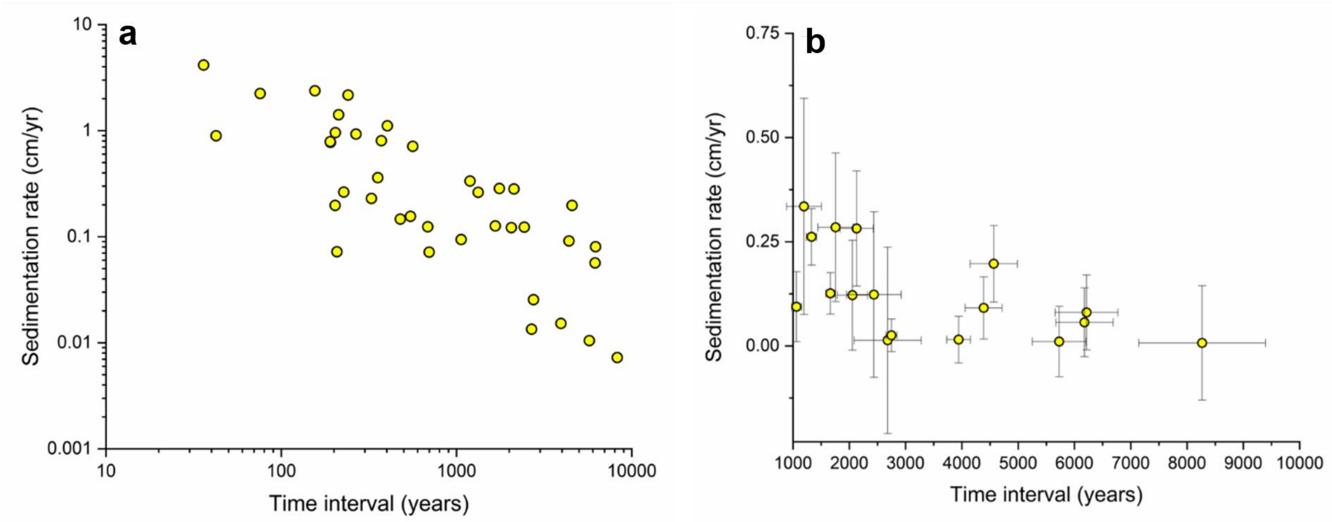

Supplementary Figure 3. **Sedimentation rates compared to timespan. a**) Sedimentation rates calculated from vertical profiles from the seasonally flooded substrates of the Solimões, Negro and Xingu rivers. **b**) Sedimentation rates variation in the millennial timespan. Each dot in panels **a** and **b** corresponds to a sedimentation rate calculated for a depth interval delimited by sediment samples with OSL or radiocarbon ages. Source data used to calculate sedimentation rates and their propagated errors are in Supplementary Data 3.

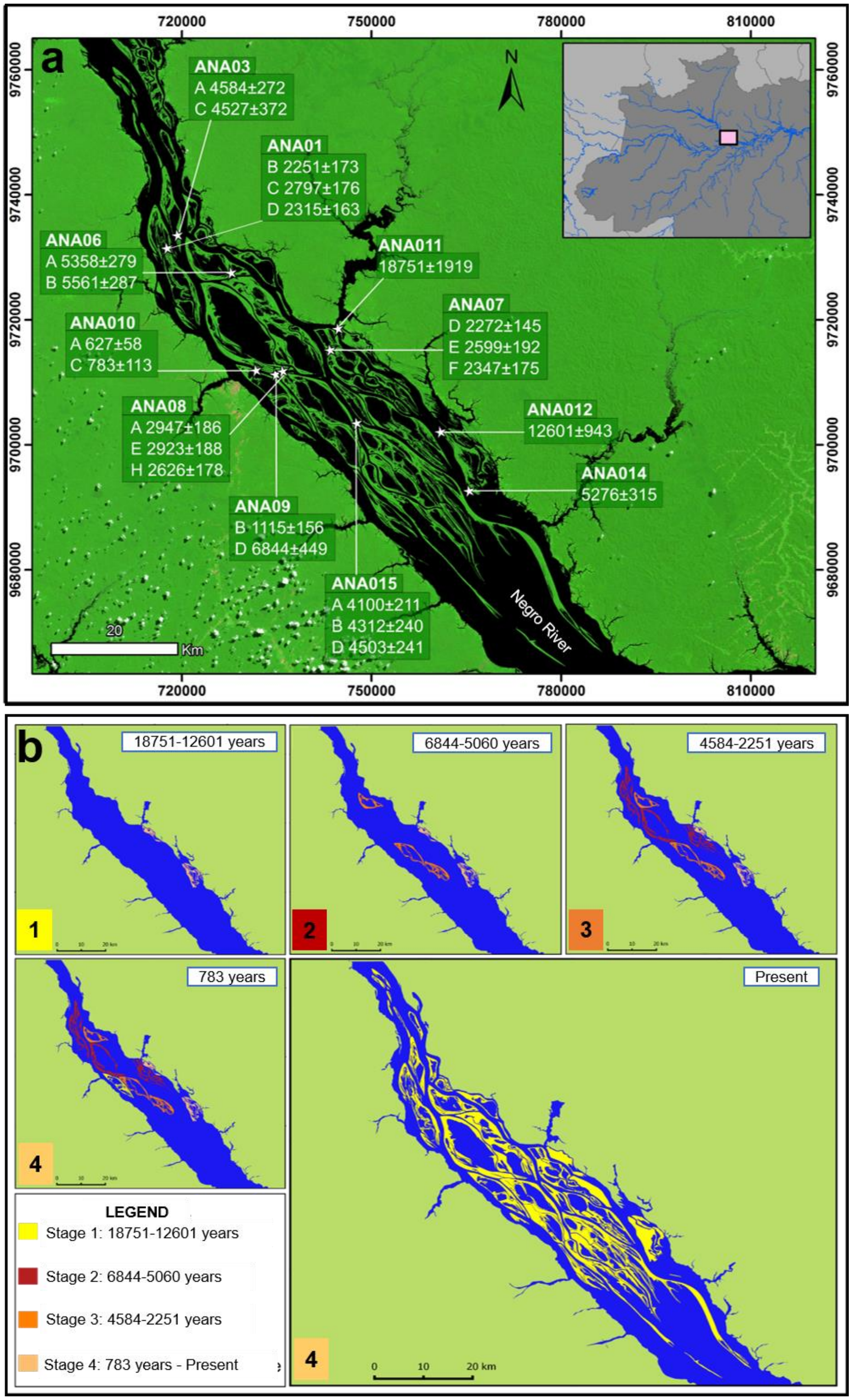

Supplementary Figure 4. OSL ages of the Anavilhanas archipelago in the Negro River. a) Sampled sites with sediment deposition ages (years) obtained in vertical profiles (see examples of vertical profiles in Supplementary Figure 5). **b**) Growth periods (Stages 1-4) of the Anavilhanas archipelago inferred from OSL ages.

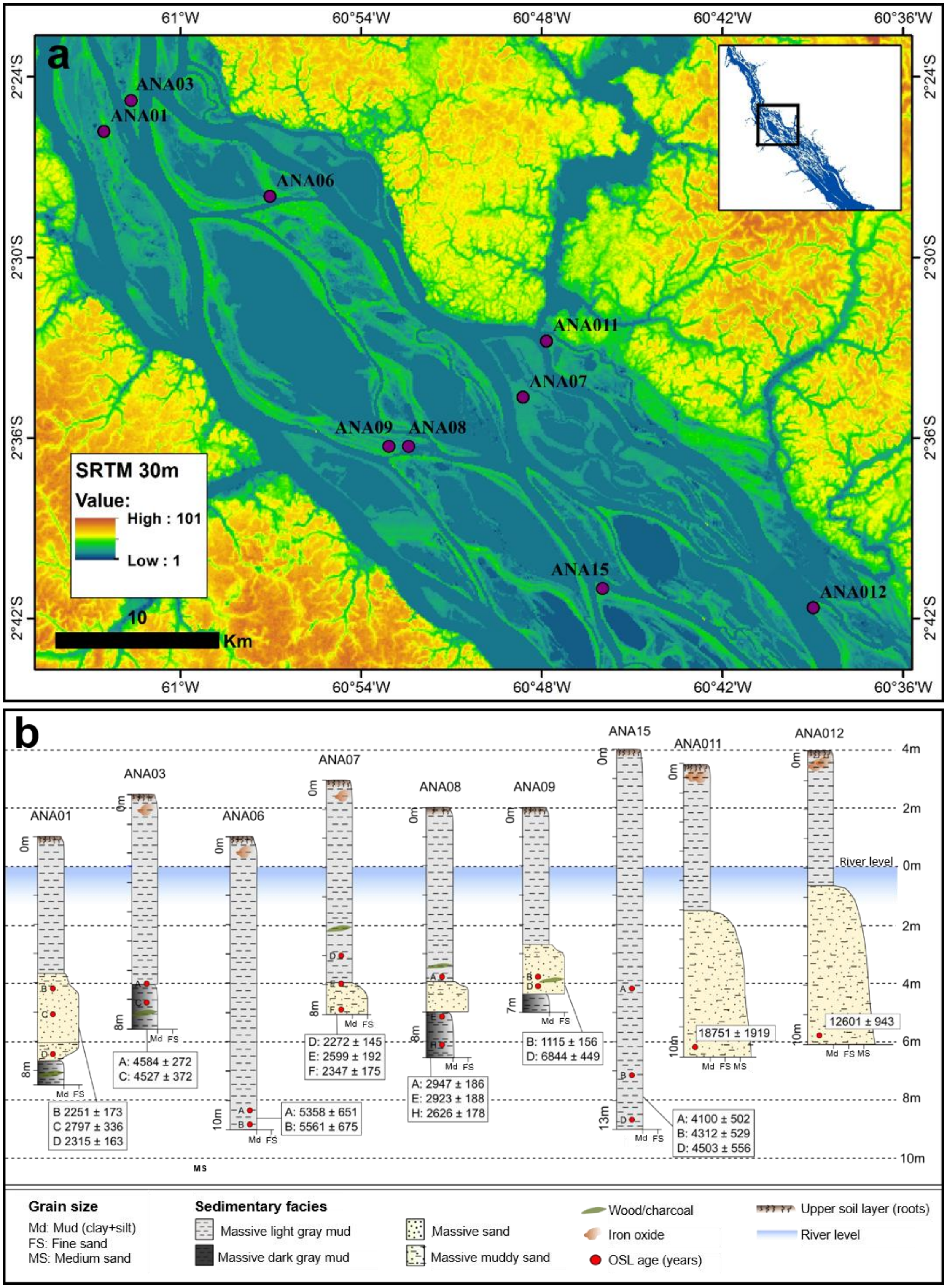

Supplementary Figure 5. Sedimentary facies columns and OSL ages of the Anavilhanas archipelago in the Negro River. a) Digital elevation model of the northern portion of the Anavilhanas archipelago, with location of substrate profiles shown in panel **b**. **b**) Sedimentary facies columns representative of the island substrates, with corresponding OSL ages.

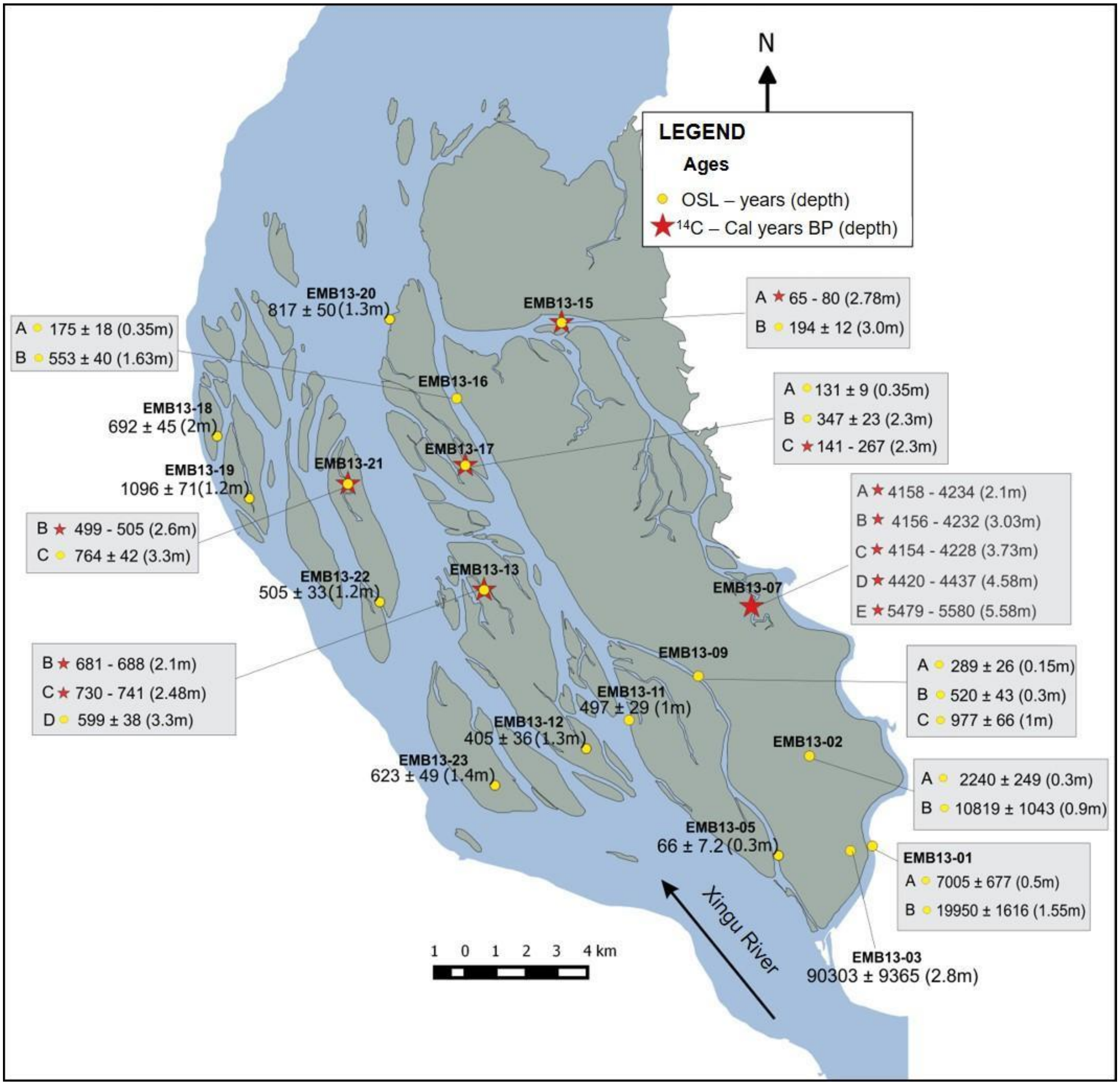

Supplementary Figure 6. OSL and radiocarbon (^{14}C) ages of the Tabuleiro do Embaubal archipelago in the Xingu River. Vertical profiles with sedimentary facies description and ages are shown in Supplementary Figures 7 and 8.

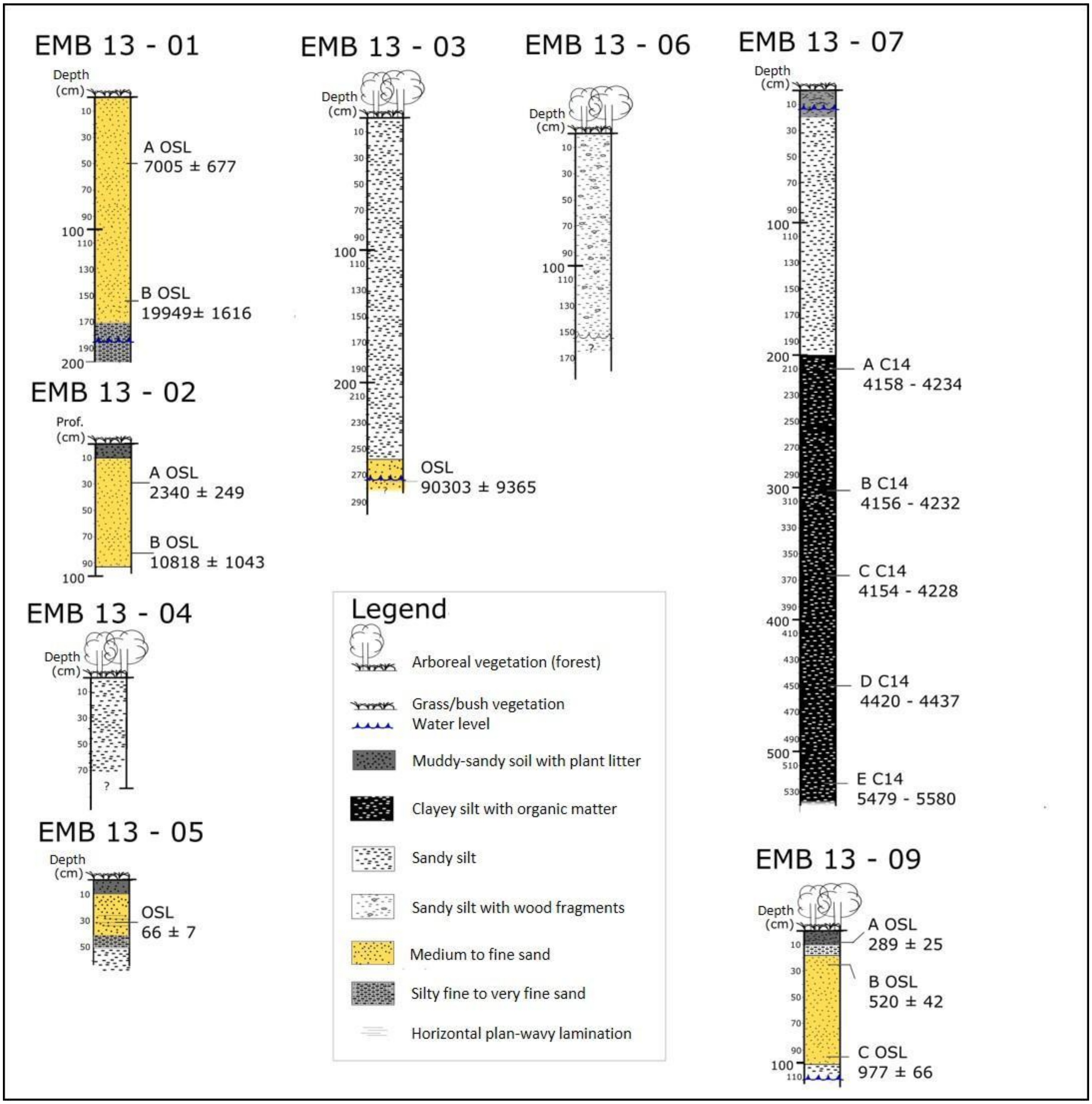

Supplementary Figure 7. Sedimentary facies columns and ages (OSL and radiocarbon) of the Tabuleiro do Embaubal archipelago in the Xingu River. OSL and radiocarbon (C14) ages are expressed in years. Location of vertical sections are shown in Supplementary Figure 6.

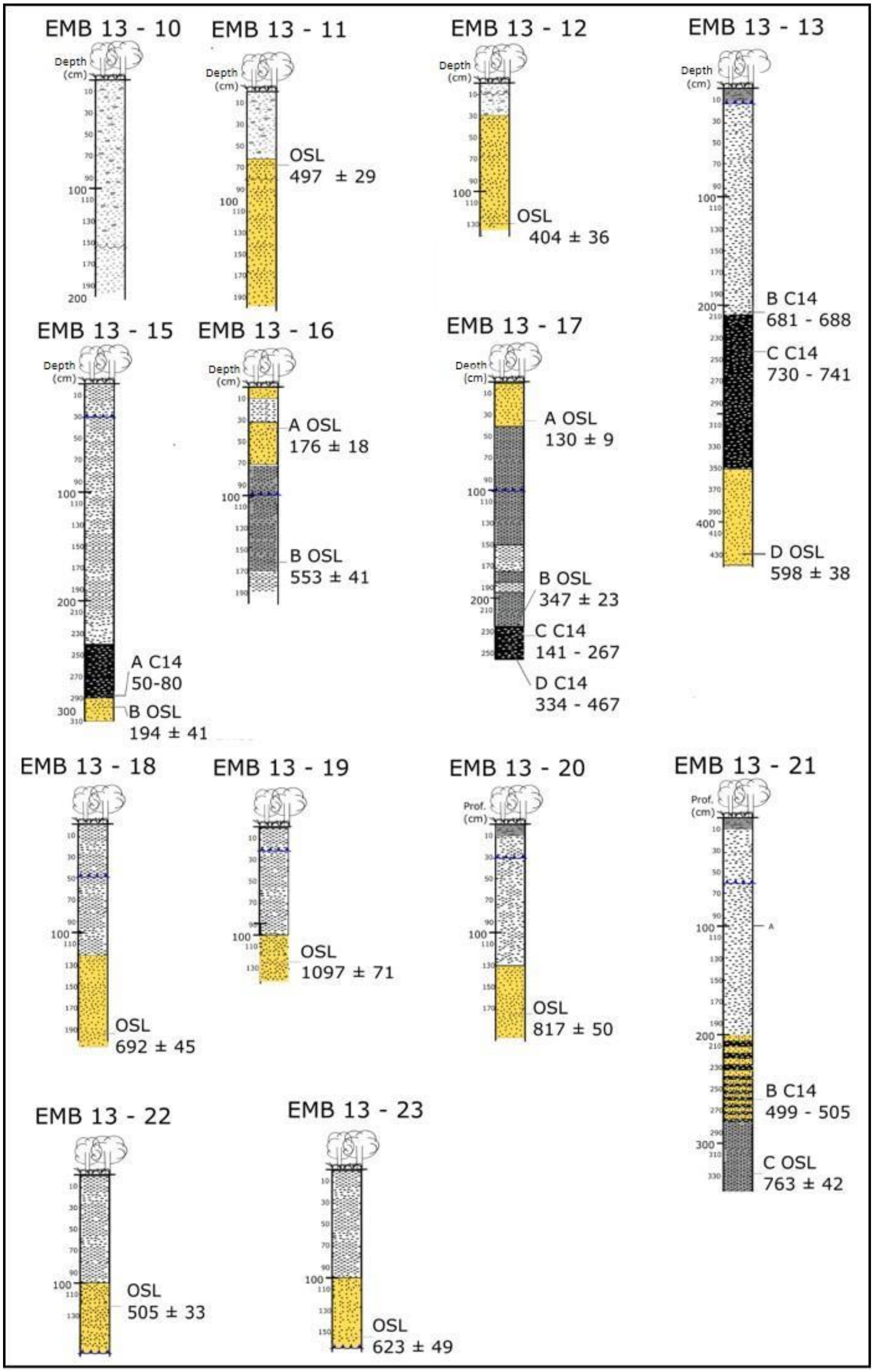

Supplementary Figure 8. Sedimentary facies columns and ages (OSL and radiocarbon) of the Tabuleiro do Embaubal archipelago in the Xingu River. OSL and radiocarbon (C14) ages are expressed in years. Location of vertical sections are shown in Supplementary Figure 6.

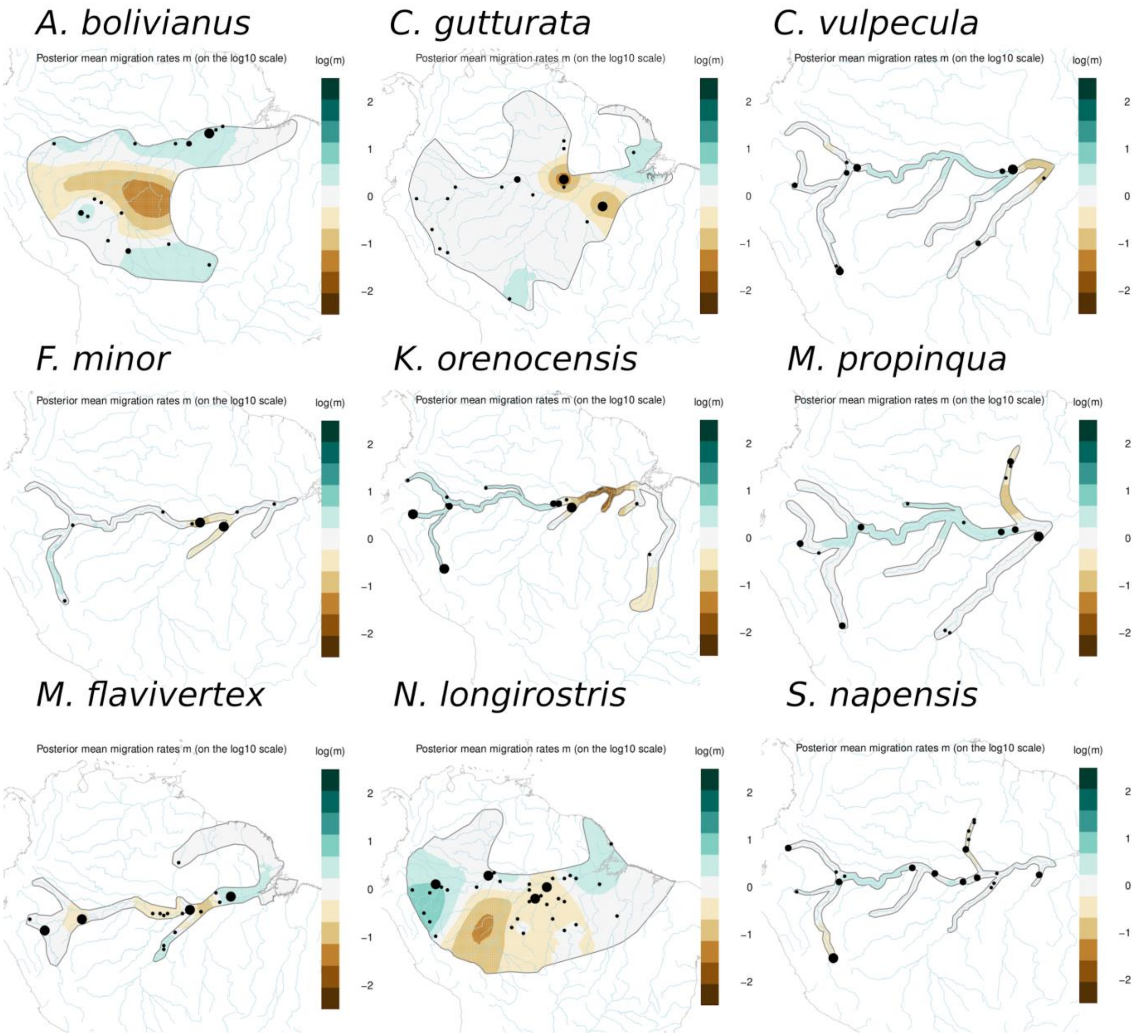

Supplementary Figure 9. Estimated Effective Migration Surface (EEMS). Black dots depict sampling sites with dot size showing the number of samples at the locality. The darker the blue, the higher than average effective migration rate and the darker the brown, the lower than average effective migration rate. Colors in the estimated effective migration surface correspond to local deviations from isolation by distance.

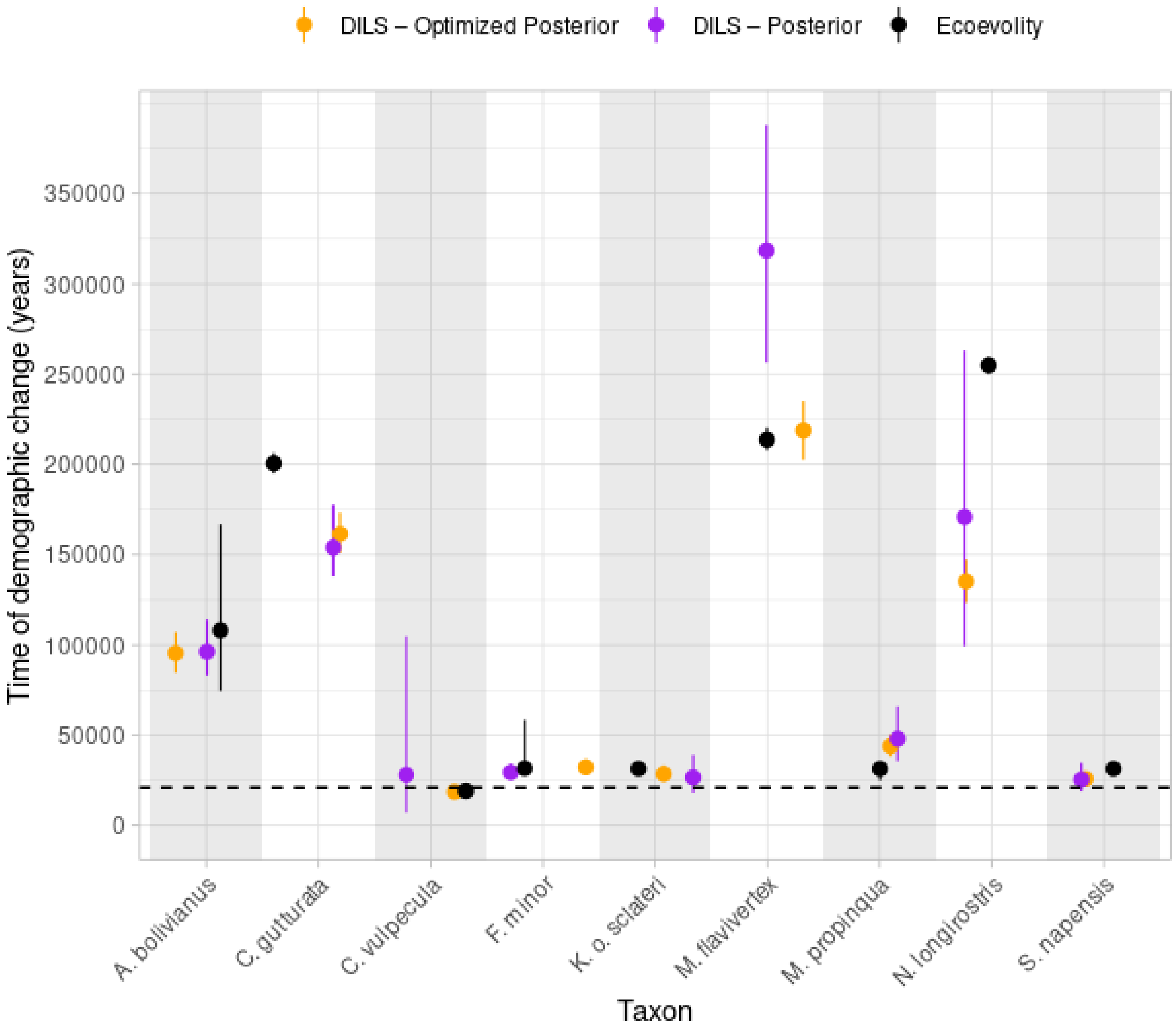

Supplementary Figure 10. Time of demographic expansion according to DILS and *Ecoevolity* analyses. Dots represent the median estimation and bars depict the 95% High Posterior Density interval. For both analyses, all available UCEs loci and samples were included, as depicted in Supplementary Data 4. Horizontal dashed line at 21 ka depicts the Last Glacial Maximum (LGM). DILS results are presented with the estimates based on the Posterior Probabilities and the Optimized Posterior from the Neural Network algorithm. Input and output files from DILS and *Ecoevolity* are available at github.com/eduardoschultz/floodplains_demographies.

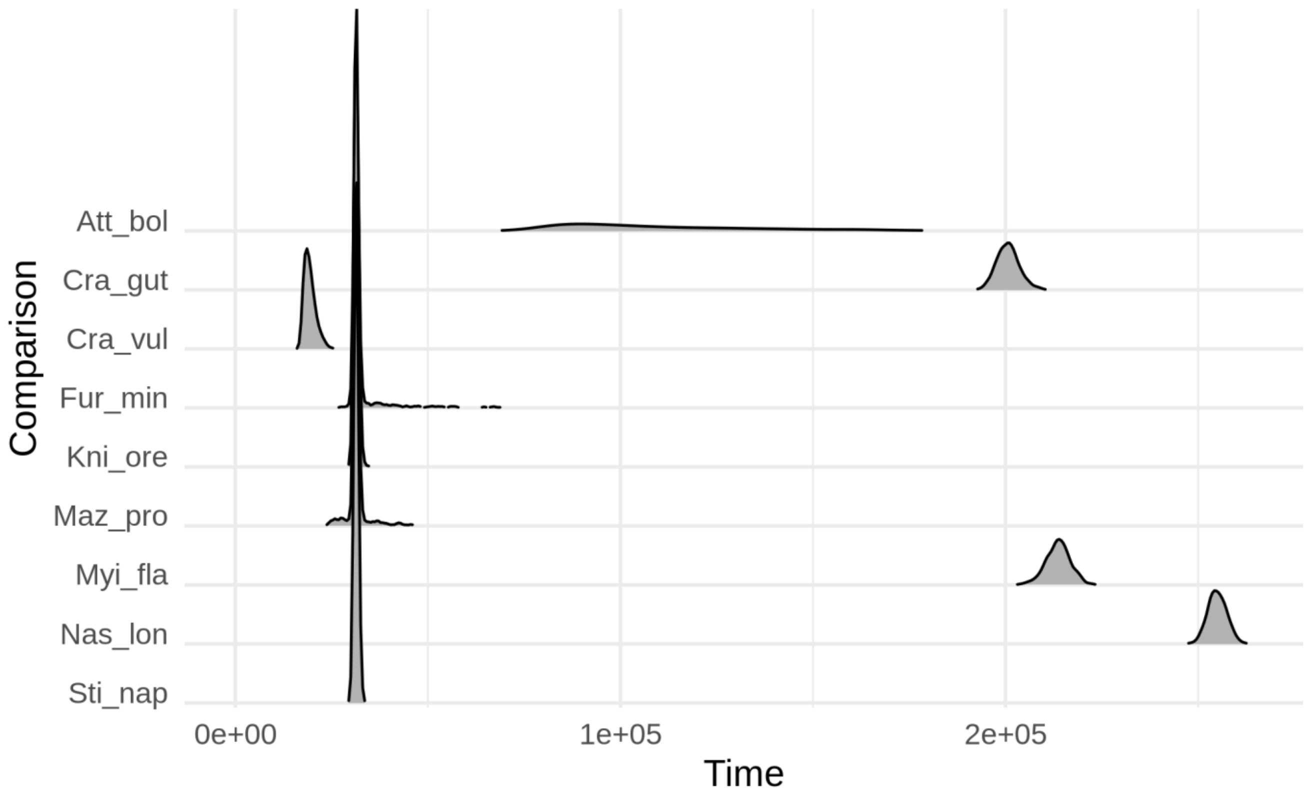

Supplementary Figure 11. Approximate marginal posterior densities of demographic change times (in years) estimated in *Ecoevolity* for each bird species. Att_bol is *A. bolivianus*, Cra_gut is *C. gutturata*, Cra_vul is *C. vulpecula*, Fur_min is *F. minor*, Kni_ore is *K. orenocensis sclateri*, Maz_pro is *M. propinqua*, Myi_fla is *M. flavivertex*, Nas_lon is *N. longirostris*, Sti_nap is *S. napensis*.

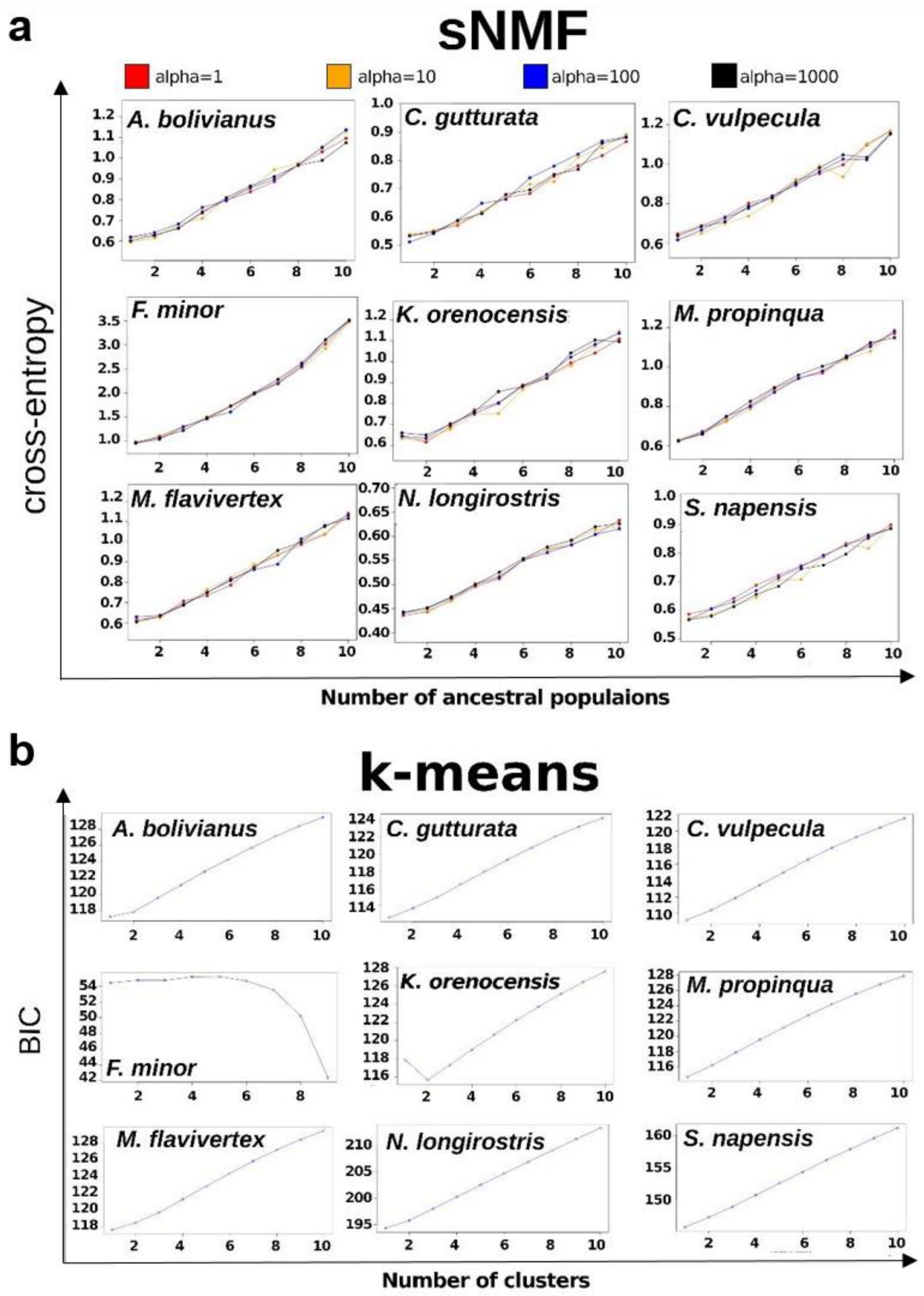

Supplementary Figure 12. Population structure analyses with sNMF (a) and k-means (b). In both analyses, the likelihood of 1 to 10 genomic populations were tested for each bird species. Lower cross-entropy and BIC values, respectively, suggest the best population arrangement based on the genomic data. For sNMF, four different alpha values were tested and results were consistent across runs. Because of the low number of samples (n=10) in *F. minor*, a maximum of nine possible populations were tested and k-means suggest each individual as a single population. Nonetheless, sNMF supports the treatment of all samples as a single population.

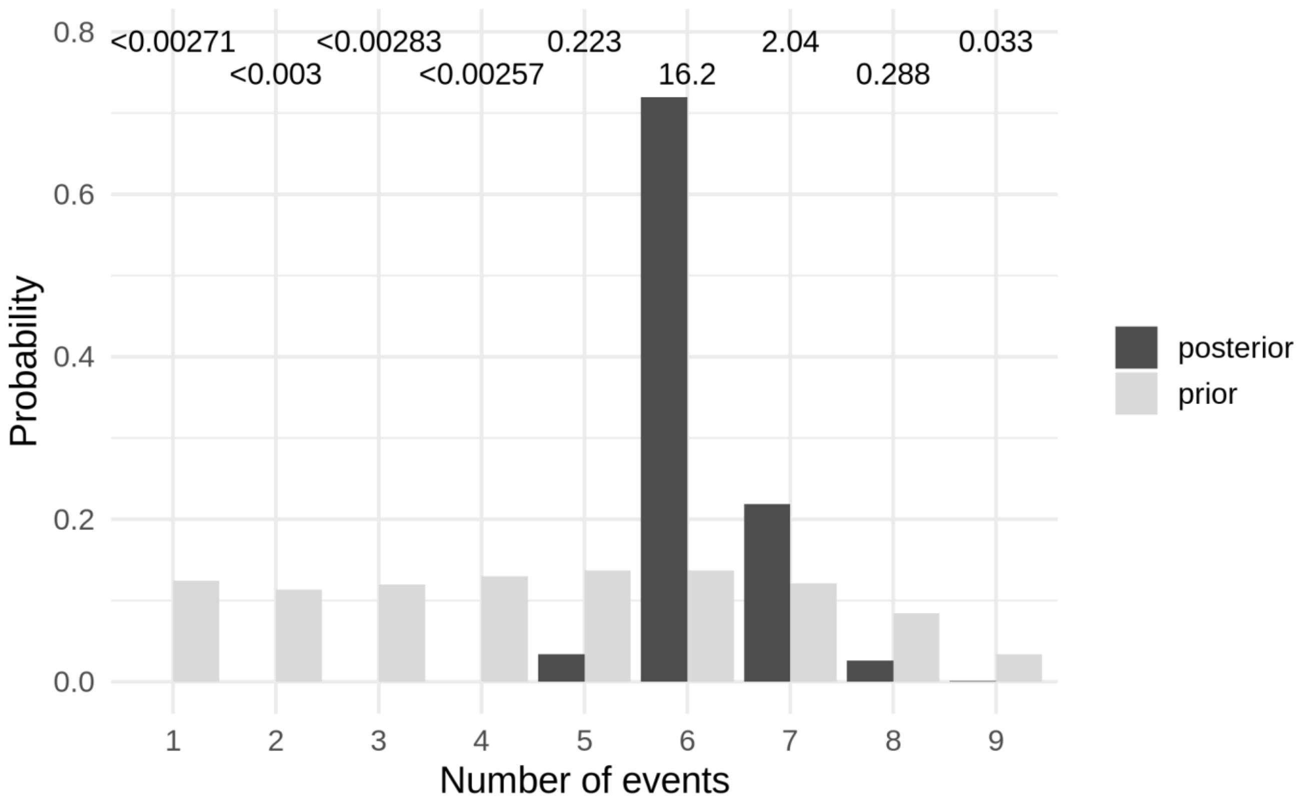

Supplementary Figure 13. Approximate prior (light bars) and posterior (dark bars) probabilities of the number of demographic expansion events across the nine studied bird species estimated in Ecoevolity. Bayes factors for each number of demographic events is given above the corresponding bars. Each Bayes factor compares the corresponding number of events to all other possible numbers of divergence events.

Supplementary Table

Supplementary Table 1. **Protocols used for equivalent dose estimation in pure quartz (OSL SAR) or polymineral (Post IR OSL SAR) aliquots.** The Post IR OSL protocols A and B aimed to measure quartz in the presence of feldspar. Seven regeneration doses (D_i) were used in all protocols: $D_1<D_2<D_3<D_4$; D_5 = 0 Gy, $D_6 = D_1$; $D_7 = D_6$. Corrected luminescence signals were calculated through the ratio between the natural or regeneration dose signal (L_i) and the test dose (Dt) signal (T_i). Dose response curves were built using signals from D_1 to D_4. The ratio between the D_5 signal and the natural signal was used to calculate recuperation. Recycling ratio was calculated through the ratio between signals from D_6 and D_1. The ratio between signals from D_6 and D_7 was used to appraise feldspar contamination in quartz aliquots (protocol OSL SAR). For this purpose, the OSL signal from D_7 was measured after an additional step of infrared stimulation at 50°C before blue light stimulation. Preheat temperatures used in the OSL SAR protocol varied in terms of the sample batch.

Step	OSL SAR (pure quartz aliquots)	Post IR OSL SAR_A (polymineral aliquots)	Post IR OSL SAR_B (polymineral aliquots)
1	Dose D_i	Dose D_i	Dose D_i
2	Preheat at 200, 220 or 240°C for 10 s	Preheat at 200°C for 10 s	Preheat at 200°C for 10 s
3	Blue stimulation at 125°C for 40 s (L_i)	Infrared stimulation at 125°C for 100 s	Infrared stimulation at 60°C for 300 s
4	Heating at 160°C	Blue stimulation at 125°C for 40 s (L_i)	Blue stimulation at 125°C for 40 s (L_i)
5	Test Dose D_t	Test Dose D_t	Test Dose D_t
6	Blue stimulation at 125°C for 40 s (T_i)	Heating at 160 °C	Heating at 200 °C
7	Blue bleach at 280°C for 40s	Infrared stimulation at 125 °C for 100 s	Infrared stimulation at 60 °C for 300 s
8	Return to step 1	Blue stimulation at 125°C for 40 s (T_i)	Blue stimulation at 125°C for 40 s (T_i)
9	-	Blue bleach at 280°C for 40 s	Blue bleach at 20°C for 40 s
10	-	Return to step 1	Return to step 1

Bioinformatics procedures

In the era of next generation sequencing and phylogenomics, bioinformatics data processing is a crucial step, with distinct approaches potentially resulting in big differences in the quality of the final data, affecting subsequent analyses. After multiple tests for our dataset, the following approach resulted in the best quality of sequences, longer sequences, higher amount of loci and faster processing. Therefore, the same approach was applied for each species to process genomic data from raw reads to phased sequence matrices and Single Nucleotide Polymorphisms (SNPs) in Phyluce (Andermann et al., 2018; Faircloth, 2016). It is based on a combination of tutorials I and II of https://phyluce.readthedocs.io/en/latest/tutorials/index.html. When not otherwise specified, each program was run using default Phyluce arguments.

Firstly, the raw reads received from Rapid Genomics were cleaned using Illumiprocessor (Faircloth, 2013). After counting the reads in each sample, one of the samples with good amount of reads (tipically more than six millions) was selected to be assembled into contigs using SPAdes (Bankevich et al., 2012). The recovered contigs were mapped to the UCE probes using phyluce_assembly_match_contigs_to_probe with minimum identity of 90%. Afterwards, one reference sequence for each locus was exported to a fasta file with get_fastas_from_match_counts to identify the sequenced loci and phyluce_assembly_get_fastas_from_match_counts to create the reference fasta. Since each sample requires one reference file with the loci sequences to map the reads, the reference file created for the assembled sample was copied for each of the other samples, correcting the names in the files and the fasta headers. Then, for each sample, the clean reads were aligned to the reference fasta using phyluce_snp_bwa_multiple_align. From the bam files resulting from the previous step, each sample was phased using phyluce_snp_phase_uces and one fasta file for each locus was created containing the two phases of all samples using phyluce_align_seqcap_align, using mafft to align the sequences (Katoh and Standley, 2013). The next step was to remove the locus name from the headers with phyluce_align_remove_locus_name_from_nexus_lines and to select only loci with sequences from all samples with phyluce_align_get_only_loci_with_min_taxa. Finally, the sequence files were trimmed with phyluce_align_get_gblocks_trimmed_alignments_from_untrimmed. SNPs dataset were obtained randomly selecting one SNP per UCE, without including missing data, with snps_from_uce_alignments.py (https://github.com/tobiashofmann88/UCE-data-management/blob/master/snps_from_uce_alignments.py).

Additionally, for each species, two mitochondrial loci (ND2 and cytb) were recovered using Geneious v7.1 (Kearse et al., 2012). Firstly, for each species, ND2 and cytb sequences were downloaded from genbank as a fasta file. In the absence of cytb or ND2 sequences available at GenBank for a species, its closest taxon was used as reference. In Geneious, the reads previously cleaned with Illumiprocessor were mapped to the reference sequence with the “map to reference” tool, using default arguments. The consensus sequence from the alignment of the reads to the reference was extracted as the sample's locus sequence. Consensus sequences for all samples were aligned into a matrix with the “multiple align” tool, selecting “Muscle alignment”. Finally, the matrix was exported as a fasta file.

References

T. Andermann, A.M. Fernandes, U. Olsson, M. Töpel, B. Pfeil, B. Oxelman, A. Aleixo, B.C. Faircloth, A. Antonelli, Allele Phasing Greatly Improves the Phylogenetic Utility of Ultraconserved Elements. *Syst. Biol.* **0**, 1–15 (2018).

A. Bankevich, S. Nurk, D. Antipov, A.A. Gurevich, M. Dvorkin, A.S. Kulikov, V.M. Lesin, S.I. Nikolenko, S. Pham, A.D. Prjibelski, A.V. Pyshkin, A.V. Sirotkin, N. Vyahhi, G. Tesler, M.A. Alekseyev, P.A. Pevzner, SPAdes: A new genome assembly algorithm and its applications to single-cell sequencing. *J. Comput. Biol.* **19(5)**, 455-477 (2012).

B.C. Faircloth, illumiprocessor: a trimmomatic wrapper for parallel adapter and quality trimming. http://dx.doi.org/10.6079/J9ILL (2013).

B.C. Faircloth, PHYLUCE is a software package for the analysis of conserved genomic loci. *Bioinformatics* **32**, 786–788 (2016).

K. Katoh, D.M. Standley, MAFFT multiple sequence alignment software version 7: Improvements in performance and usability. *Mol. Biol. Evol.* **30(4)**, 772-780 (2013).

M. Kearse, R. Moir, A. Wilson, S. Stones-Havas, M. Cheung, S. Sturrock, S. Buxton, A. Cooper, S. Markowitz, C. Duran, T. Thierer, B. Ashton, P. Meintjes, A. Drummond, Geneious Basic: An integrated and extendable desktop software platform for the organization and analysis of sequence data. *Bioinformatics* **28(12)**, 1647-1649 (2012).
