# Supplementary material for: Rainfall and sea level drove the expansion of seasonally flooded habitats and associated bird populations across Amazonia
Source: Nat Commun. 2022 Aug 23;13:4945. doi: 10.1038/s41467-022-32561-0 (PMC9399099; doi:10.1038/s41467-022-32561-0)
Supplement: Supplementary file 7 — Reporting Summary [file 41467_2022_32561_MOESM7_ESM.pdf]

nature portfolio

Corresponding author(s): Andre Oliveira Sawakuchi

Last updated by author(s): July 14, 2022

Reporting Summary

Nature Portfolio wishes to improve the reproducibility of the work that we publish. This form provides structure for consistency and transparency in reporting. For further information on Nature Portfolio policies, see our Editorial Policies and the Editorial Policy Checklist.

Statistics

For all statistical analyses, confirm that the following items are present in the figure legend, table legend, main text, or Methods section.

n/a	Confirmed	
☐	☒	The exact sample size (*n*) for each experimental group/condition, given as a discrete number and unit of measurement
☐	☒	A statement on whether measurements were taken from distinct samples or whether the same sample was measured repeatedly
☒	☐	The statistical test(s) used AND whether they are one- or two-sided<br>*Only common tests should be described solely by name; describe more complex techniques in the Methods section.*
☒	☐	A description of all covariates tested
☒	☐	A description of any assumptions or corrections, such as tests of normality and adjustment for multiple comparisons
☐	☒	A full description of the statistical parameters including central tendency (e.g. means) or other basic estimates (e.g. regression coefficient) AND variation (e.g. standard deviation) or associated estimates of uncertainty (e.g. confidence intervals)
☐	☒	For null hypothesis testing, the test statistic (e.g. *F*, *t*, *r*) with confidence intervals, effect sizes, degrees of freedom and *P* value noted<br>*Give P values as exact values whenever suitable.*
☐	☒	For Bayesian analysis, information on the choice of priors and Markov chain Monte Carlo settings
☐	☒	For hierarchical and complex designs, identification of the appropriate level for tests and full reporting of outcomes
☒	☐	Estimates of effect sizes (e.g. Cohen's *d*, Pearson's *r*), indicating how they were calculated

Our web collection on statistics for biologists contains articles on many of the points above.

Software and code

Policy information about availability of computer code

Data collection	Genomic data collection was outsourced to Rapid Genomics, Florida, USA
Data analysis	Phyluce Pipeline (Faircloth, 2016), Illumiprocessor 2.0.9 (Faircloth, 2013), SPAdes 3.12.0-1 (Bankevich et al., 2012), mafft v7.471 (Katoh and Standley, 2013), jModeltest 2.1.10 (Darriba et al., 2012),Geneious 7.1 (Kearse et al., 2012), LEA 2.0 and ADEGENET 2.1.5 R packages (Frichot et al., 2014; Jombart et al., 2011; R Core Team), EEMS 0.0.0.9000 (Petkova et. al., 2017), Qgis 3.14 (QGIS Development Team, 2022) snps_from_alignments.py (https://github.com/tandermann/UCE-data-management/blob/master/snps_from_uce_alignments.py), BEAST v2.5.2 (Bouckaert et al., 2019), DILS (Fräisse et al., 2021), Ecoevolity 0.3.2 (Oaks, 2019).

For manuscripts utilizing custom algorithms or software that are central to the research but not yet described in published literature, software must be made available to editors and reviewers. We strongly encourage code deposition in a community repository (e.g. GitHub). See the Nature Portfolio guidelines for submitting code & software for further information.

Data

Policy information about availability of data

All manuscripts must include a data availability statement. This statement should provide the following information, where applicable:

- Accession codes, unique identifiers, or web links for publicly available datasets
- A description of any restrictions on data availability
- For clinical datasets or third party data, please ensure that the statement adheres to our policy

All data needed to evaluate the conclusions in the paper are present in the paper and/or in the Supplementary Material. All sequence data is deposited in GenBank. The genomic reads used in this study are deposited in the National Center for Biotechnology Information Sequence Read Archive under the project PRJNA819080. The input and config files, as well as the results of demographic analyses, are available at github.com/eduardoschultz/floodplains_demographies.

Field-specific reporting

Please select the one below that is the best fit for your research. If you are not sure, read the appropriate sections before making your selection.

☐ Life sciences ☐ Behavioural & social sciences ☒ Ecological, evolutionary & environmental sciences

For a reference copy of the document with all sections, see nature.com/documents/nr-reporting-summary-flat.pdf

Ecological, evolutionary & environmental sciences study design

All studies must disclose on these points even when the disclosure is negative.

Study description	We sequenced 2500 Ultraconserved Elements (UCEs) and the mitochondrial genome of the studied taxa, and used three different approaches to infer their demographic history. With a subsample of the UCEs and two mitochondrial markers we recovered the population size dynamics through time with Extended Bayesian Skyline Plots (EBSP) in BEAST 2.5. To compare with EBSP results, in DILS we used the whole genomic dataset to test the probability of different demographic models: population expansion, contraction or stability. Finally, with Ecoevolity we tested the probability of the recovered demographic expansions in all species having occurred concomitantly.
Research sample	We focused on sampling taxa with broad distribution across the Amazonian floodplains and specialized on distinct floodplain environments. For each taxon, we searched for samples available at Scientific Collections in Brazil and the USA to cover as much as possible the taxon's whole distribution. We sampled 210 individuals belonging to 9 bird species specialized in Amazonian seasonally flooded habitats: Cranioleuca vulpecula, Furnarius minor, Knipolegus orenocensis, Mazaria propinqua, Stigmatura napensis, Attila bolivianus, Cranioleuca gutturata, Myiopagis flavivertex, and Nasica longirostris. All individuals have associated voucher specimens. Genomic sampling was based on a probe set targeting Ultraconserved Elements. We obtained an average of 2233 UCE loci and 1574 SNPs per individual.
Sampling strategy	For each of the 9 species, indiduals samples were chosen aiming to cover their known geographic distribution along seasonally flooded habitis in Amazonia.
Data collection	Fresh tissue samples were obtained through loans from Scientific Collections. All field samplings in Brazil that contribute specimens for Scientific Collections are regulated by the Environmental Ministry / Brazilian Government through Collecting Permits issued by ICMBio/MMA. Eduardo D. Schultz extracted DNA from the tissues with QIAGEN Dneasy Blood and Tissue kits in laboratories at INPA and AMNH, prepared the plates and sent the extracts to Rapid Genomics for sequence capture following Brazilian legislation. All samples collected outside Brazil were loaned from US institutions following their loan policies. Sequences from each species were processed independently from raw reads to final UCE alignments through the Phyluce pipeline by Eduardo D. Schultz.
Timing and spatial scale	Not applicable. Specimens were deposited in Biological Collections, spatial scale is the Amazonian floodplains.
Data exclusions	All obtained sequence data that passed through quality check was used.
Reproducibility	All raw reads are deposited in Genbank and all analytical procedures are available through Github. All data deposited in Genbank have high read counts and have been processed successfully multiple times from first data explorations to final processing. On Github the input and config files contain all the set of parameters used for the analyses, ensuring reproducibility.
Randomization	For the EBSP demographies, we used 50 random UCE loci for each species. Before final analyses, initial tests were made using different random UCEs and using the same priors and parameters of the present analyses, results were highly consistent. Moreover, each EBSP run starts with a random seed and, for all species, runs with different seeds recovered the same results. In DILS, although we provide all available loci as input, the program uses 1000 random loci to calculate the statistics. For all species, different runs, where different random loci were chosen to calculate the statistics were performed to check for consistency, and also resulted in highly consistent results.
Blinding	Blinding is not applicable because we must know which taxon we are working with. However data processing and demographic analyses were performed independently for each taxon using the same approaches and without informing the habitat affinities of the taxa, all analyses recovered the distinct demographic histories between floodplain forest and island-specialists birds.

Did the study involve field work? ☐ Yes ☒ No

Reporting for specific materials, systems and methods

We require information from authors about some types of materials, experimental systems and methods used in many studies. Here, indicate whether each material, system or method listed is relevant to your study. If you are not sure if a list item applies to your research, read the appropriate section before selecting a response.

Materials & experimental systems

n/a	Involved in the study	
☒	☐	Antibodies
☒	☐	Eukaryotic cell lines
☒	☐	Palaeontology and archaeology
☐	☒	Animals and other organisms
☒	☐	Human research participants
☒	☐	Clinical data
☒	☐	Dual use research of concern

Methods

n/a	Involved in the study	
☒	☐	ChIP-seq
☒	☐	Flow cytometry
☒	☐	MRI-based neuroimaging

Animals and other organisms

Policy information about studies involving animals; ARRIVE guidelines recommended for reporting animal research

Laboratory animals	Not applicable
Wild animals	Tissue samples were obtained as loans from Biological Collections of the following institutions: Instituto Nacional de Pesquisas da Amazonia (Manaus, Brazil), Museu Paraense Emilio Goeldi (Belem, Brazil), American Museum of Natiral History, Academy of Natural Sciences of Philadelphia, and Louisiana State University Museum of Natural Science.
Field-collected samples	The study involved tissue samples loaned from Biological Collections
Ethics oversight	Collecting Permits in Brazil under which specimens were originally collected were issued by ICMBio/ Ministry of Environment / Brazilian Federal Government. No further ethical approval was necessary. Tissue samples are housed in Brazilian and American Scientific Collections following all regulations.

Note that full information on the approval of the study protocol must also be provided in the manuscript.
